# Long-term impact of Amazon river runoff on northern hemispheric climate

**DOI:** 10.1038/s41598-017-10750-y

**Published:** 2017-09-08

**Authors:** S. Jahfer, P. N. Vinayachandran, Ravi S. Nanjundiah

**Affiliations:** Centre for Atmospheric and Oceanic Sciences, Indian Institute of Science, Bangalore, India

## Abstract

Amazon discharges a large volume of freshwater into the ocean, yet its impact on climate is largely unknown. Climate projections show that a warmer northern tropical Atlantic Ocean together with a warmer equatorial Pacific lead to extreme droughts in the Amazonia, considerably reducing the Amazon runoff. Here we present results from coupled model simulations and observations on the climatic response to a significant reduction in Amazon runoff into the Atlantic Ocean. Climate model simulation without Amazon runoff resulted in cooler equatorial Atlantic, weakening the Hadley cell and thereby the atmospheric meridional cells. Consequently, the extratropical westerlies turned weaker, leading to prevalent negative North Atlantic Oscillation (NAO) like climate, similar to the observed anomalies during Amazon drought years. This study reaffirms that spatial signature of NAO is in part driven by sea surface temperature (SST) anomalies in the tropical Atlantic. Winters of northern Europe and eastern Canada turned cooler and drier whereas southern Europe and the eastern United States experienced warmer and wetter winters without Amazon runoff. Significant warming over the Arctic reduced the local sea-ice extent and enhanced the high latitude river runoff. More importantly, our simulations caution against extreme exploitation of rivers for its far-reaching consequences on climate.

## Introduction

Amazon river discharges about 6.6 × 10^3^ Km^3^ of freshwater into the ocean annually, roughly about 17% of global runoff and ~50% of Atlantic runoff^1^. Seasonally, maximum rainfall over Amazonia occurs in the northern hemispheric winter (December to February) and spring (March to May) when the deep convection is highest^2^. The runoff maxima are observed during May to June, lagging the rainfall by 2 to 3 months^1^. Variability in this runoff is predominantly controlled by the rainfall over the Amazon basin (2000 mm year^﻿﻿-1﻿^), which in turn is closely bound to the phase of sea surface temperature (SST) in the equatorial Pacific^3^; annual mean rainfall over Amazonia is lower than normal during an El Niño event^4^. SST variability in the tropical Atlantic can also affect the rainfall over the Amazon basin^5^. A warmer northern tropical Atlantic ocean along with a cooler southern counterpart generates a dipole SST pattern across the thermal equator that pushes the ITCZ northwestward, away from Amazonia, leading to drier conditions^6^.

Some of the recent historical drought events in the Amazonia (for e.g., 2005 and 2010) were independent of El Niño but driven by unusual warming in the northern tropical Atlantic^6^. There is an increased risk of prolonged droughts over Amazonia owing to global warming related increase in the frequency of El Niño events that will potentially lower Amazon runoff into the Atlantic^7^. At present, more than a hundred dams have been constructed across the Amazon tributaries^8^. In the next few decades, an additional hundred or more such hydropower dams will be constructed as per the present proposals^9^. This will result in a massive reduction of freshwater reaching the equatorial Atlantic Ocean and there will be only a few free flowing tributaries left in the Amazonia^9^. Furthermore, state-of-the-art climate model projections for 21st century expose the risk of strengthening of drought events in the Amazonia^10^ owing to prolonged dry season^11^; implying that scenarios with considerably reduced runoff into the Atlantic is likely to occur more frequently in the future. In wake of these revelations, we have addressed how the climate system would respond to a considerable reduction in Amazon runoff, using a fully coupled climate model.

Ocean model simulations have demonstrated that, in the absence of Amazon runoff, a meridional dipole sea surface temperature (SST) anomaly develops in the equatorial Atlantic^12^. This meridional SST seesaw (cooling to the south and warming to the north of the equator) is driven by changes in Atlantic meridional overturning circulation (AMOC) in many freshwater hosing experiments (for e.g., ref. 13), and is used as a proxy for the strength of the AMOC. Atmospheric impact of this SST anomaly would be subsequent changes in the large-scale meridional atmospheric circulation^14^. Using climate model simulations, we have shown that global river runoff can modulate prominent air-sea interactions in the tropics including El Niño Southern Oscillation (ENSO) and Indian monsoon^15^. Yet, our understanding of the impact of runoff from river systems such as the Amazon is in its infancy. The present study aims at a comprehensive understanding of the role of Amazon runoff into the Atlantic on major modes of climate variability in the northern hemisphere, such as North Atlantic Oscillation (NAO)^16^.

NAO is the manifestation of meridional sea level pressure (SLP) fluctuations between subtropical high and subpolar low pressure centers, controlling the climate variability over Europe, Greenland, North America, and the Mediterranean^14, 16^. The negative phase of NAO (NAO−) is known to be associated with weaker meridional pressure gradient in the northern Atlantic, diminished westerlies to the northern Europe, and a weaker AMOC^17, 18^. Atmospheric modeling studies have demonstrated that the tropical Atlantic SST anomalies can drive low-frequency NAO variability^19^. Amazon runoff that streams into the equatorial Atlantic, stratifies the upper ocean^12^, leading to changes in air-sea interaction, followed by changes in tropical Atlantic SST. Does this SST anomaly triggered by Amazon runoff have the potential to modulate the strength of meridional pressure gradient in the extra-tropics and regulate NAO variability? This is important as NAO is the most prominent mode of climate variability in the northern hemisphere. To understand this, coupled model experiments were carried out by intercepting Amazon river runoff into the Atlantic ocean in order to assess the impact of prolonged droughts over Amazonia. Using coupled model simulations that include and exclude Amazon runoff (see methods), we find that significant reduction in Amazon runoff could result in teleconnections that severely affect climate over North America and Europe via a change in phase of NAO.

## Results

### Oceanic response

**Figure 1:**
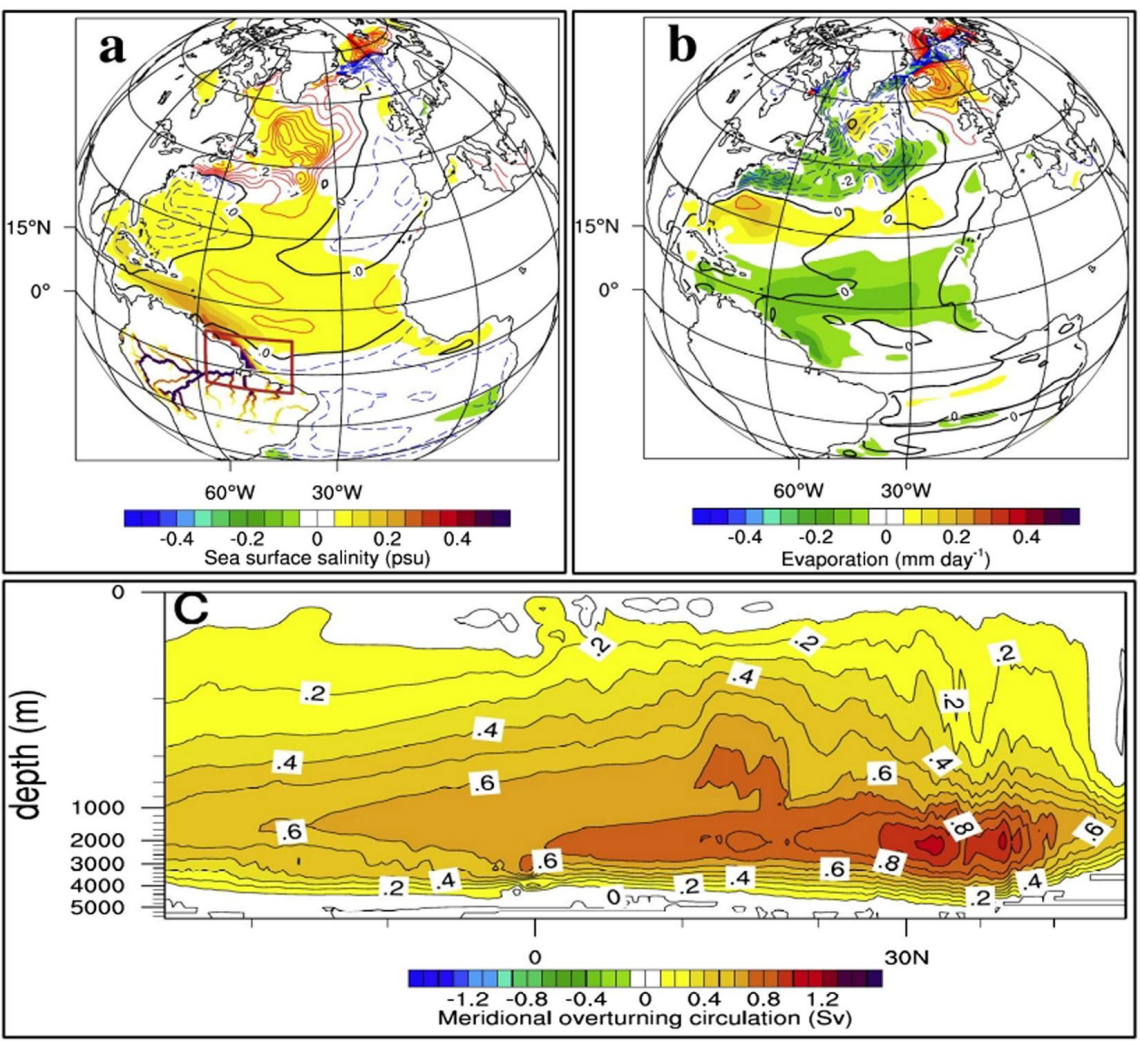
Oceanic response in the absence of Amazon runoff. Amazon river network in the RTM model is shown in (**a**) with a box where the Amazon runoff into the ocean is intercepted in the coupler. Mean difference (AMZ minus CR (AMZ-CR)) in oceanic parameters over the 100–200 year model period. (**a**) Shading represents annual mean SSS (psu) overlaid with contours representing DJF mean SST difference (°C) with black, red and blue contours indicating zero, positive, and negative anomalies respectively. (**b)** DJF mean difference in the evaporation (mm day^−1^, shaded) and sensible heat flux (contour with the same color pattern as (**a**)). For (**b**), negative anomalies show that the ocean gains heat (reduced heat loss). (**c)** Annual mean difference in meridional overturning circulation (MOC) in the Atlantic with a contour interval of 0.2 Sv. Positive MOC represents clockwise circulation. Contours and shades in (**a**) and (**b**) exceed 90% confidence level, calculated using students t-test. Plots were generated using an open software NCAR Command Language (NCL, Version 6.3.0) [Software] (2016). Boulder, Colorado: UCAR/NCAR/CISL/TDD. http://dx.doi.org/10.5065/D6WD3XH5. Background map used is the satellite projection provided by NCL.

Freshwater from Amazon river discharges into the northwestern tropical Atlantic, the effect of which is a low saline, highly stratified upper ocean^15^. Atlantic is the most sensitive ocean basin to freshwater perturbation from rivers, in terms of changes in salinity and temperature^15^. In the last 100 years of the model run, annual mean sea surface salinity (SSS) anomalies (difference in SSS between simulation without Amazon runoff (AMZ) and the reference run (CR); see methods) due to shutting off Amazon runoff, are predominantly to the north of the equator (Fig. 1a, shading). The SST response (Fig. 1a) is marked by a surface cooling (blue contour) of 0.2 °C in the southern tropical Atlantic and a mild warming (red contour) to the north during DJF, with zero contour (black thick line) along 5°N. SST anomaly in the northern Atlantic (Fig. 1a) with cooling centered around 30°N and 35°N (~0.2 °C) in the northwest and northeast Atlantic respectively and a warming (0.3 °C) centered around 50°N, exhibits striking resemblance to the tripole pattern of SST anomalies associated with NAO^19^. Changes in oceanic evaporation (Fig. 1b, shading) and sensible heat (Fig. 1b, contour) are the outcome of complex air-sea interaction in the absence of Amazon runoff (explained in the Discussed section). In the absence of Amazon runoff, the AMOC strengthened with a maximum increase of ~1.2 Sverdrup (Sv) at a depth of around 2000 m (Fig. 1c).

**Figure 2:**
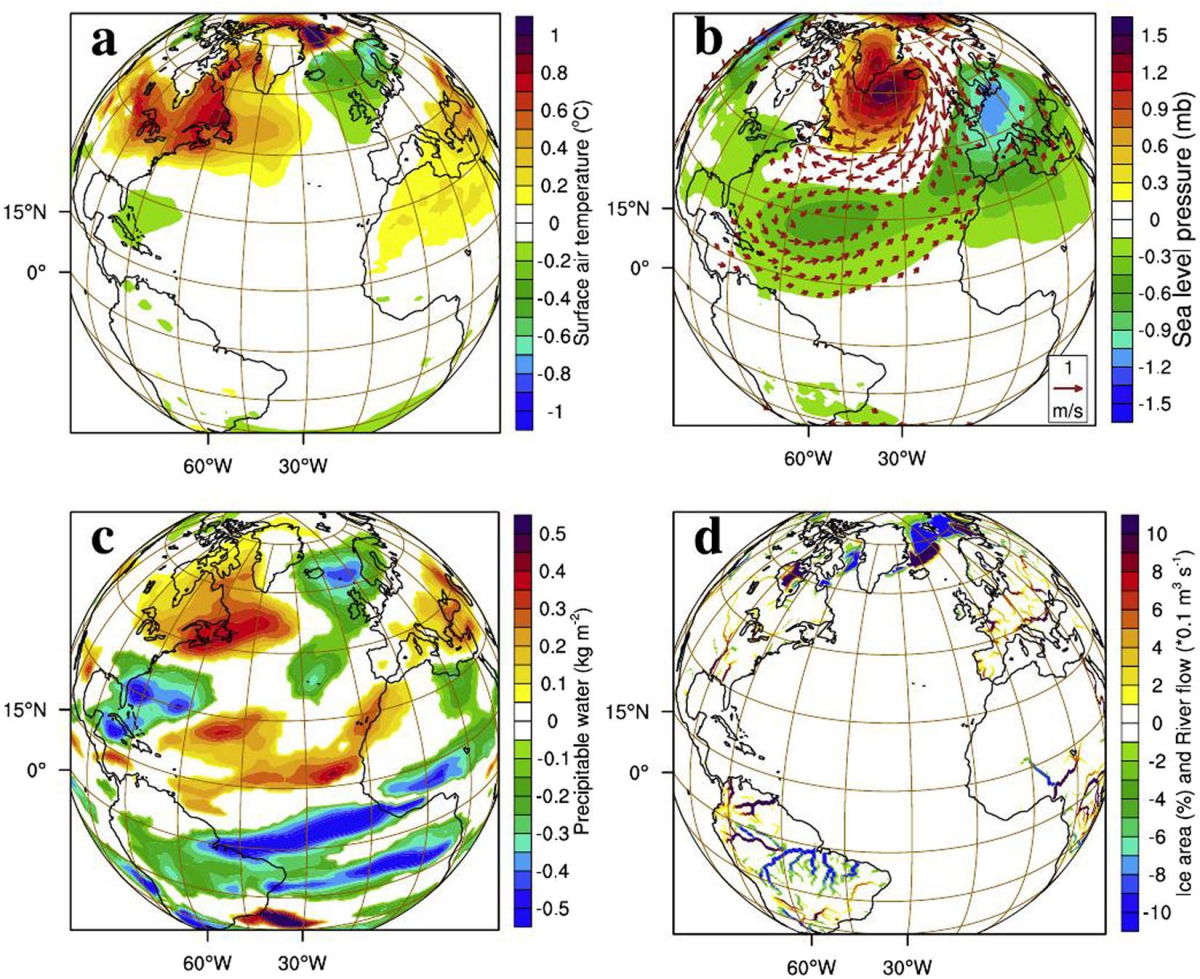
Atmospheric response in the absence of Amazon runoff. Mean winter (DJF) response (AMZ-CR) of (**a**), surface air temperature (°C), (**b**) sea level pressure (mb) and winds (m s^−1^) with a reference vector of 1 m s^−1^, (**c**), precipitable water (kg m^−3^) and **d**, river runoff (×10^−1^ m ^3^ s^−1^) in the land model and percentage change in ice area (%). Regions, where the responses are significant at 90% confidence level, are plotted. Plots were generated using an open software NCL (Version 6.3.0) [Software] (2016). Boulder, Colorado: UCAR/NCAR/CISL/TDD. http://dx.doi.org/10.5065/D6WD3XH5. Background map used is the satellite projection provided by NCL.

**Figure 3:**
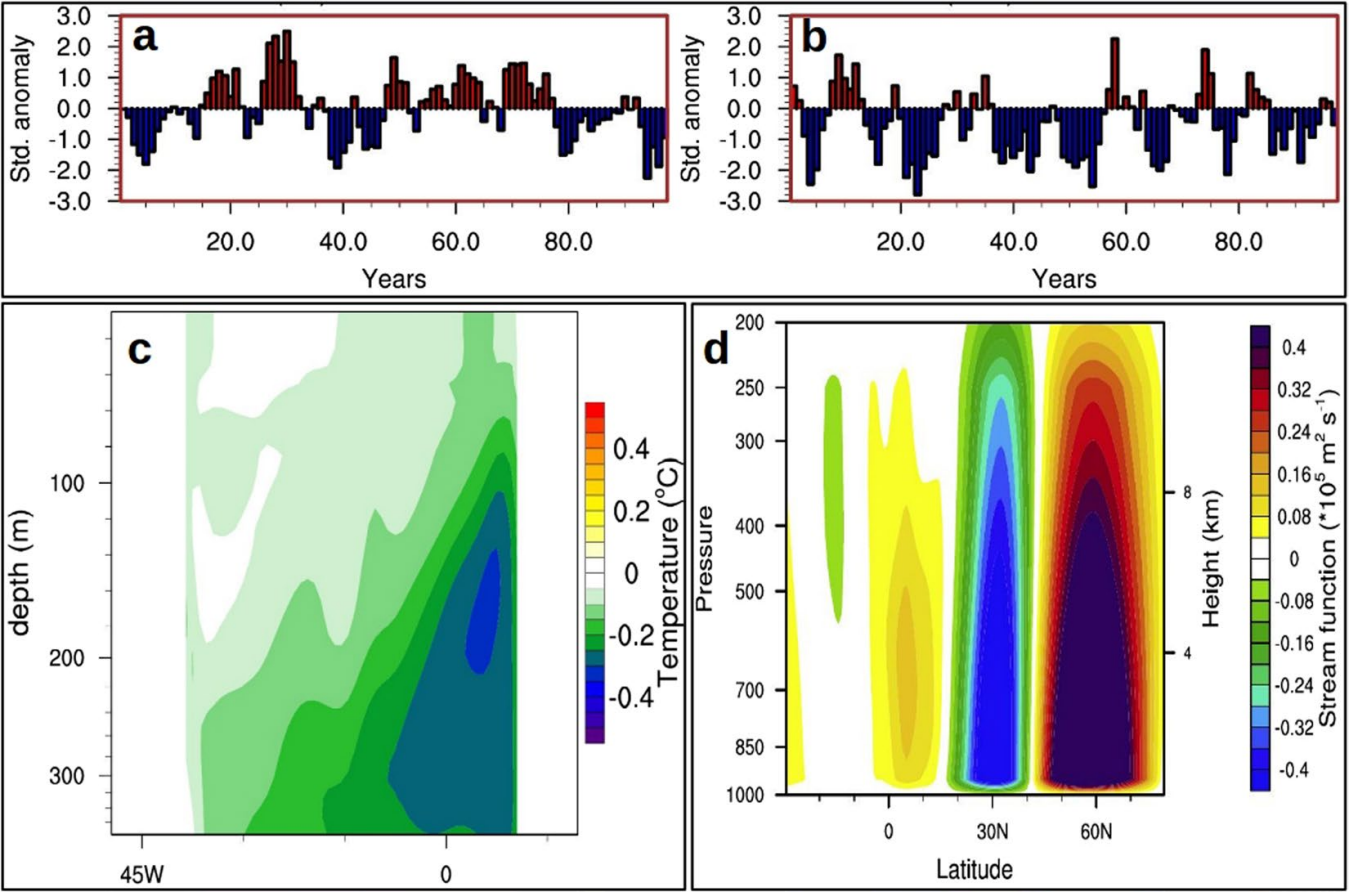
Negative phase of NAO and its impacts: I. **(a**) 100–200 years CR NAO index based on 4-year running average of the normalized DJF sea level pressure gradient between subtropical high pressure center (35°N–55°N, 20°W–20°E) and subpolar low pressure center (60°N–80°N, 20°W–20°E) in the Atlantic. (**b**) Same as (**a**) but for AMZ. (**c**) Mean upwelling in the southern tropical Atlantic shown as the difference in DJF temperature profile (°C) averaged over 20°S–15°S band. Cooler regions are close to the eastern coast of Africa. (**d**) Mean DJF difference in meridional stream function (×10^5^ m^2^ s^−1^) for the Atlantic region (70°W–20°E, 30°S–80°N). Positive anomalies show reduced ascend and negative anomalies show reduced descend. Reduced ascend over the equatorial low and subpolar high along with reduced descend over subtropical high clearly indicate a weaker atmospheric meridional circulation in the Atlantic sector. Shaded regions are significant at 90% confidence level using t-test. Plots were generated using an open software NCL (Version 6.3.0) [Software] (2016). Boulder, Colorado: UCAR/NCAR/CISL/TDD. http://dx.doi.org/10.5065/D6WD3XH5. Background map used is the satellite projection provided by NCL.

Winter-time atmospheric signals in response to blocking of Amazon runoff resemble the anomalous surface air temperature (Fig. 2a), surface pressure and 850 mb wind (Fig. 2b) patterns during NAO−. Subtropical high pressure belt centered around 30°N, stretching over the Atlantic and adjacent landmass, lowered by about 0.5 mb, leading to a weaker than normal high pressure belt (Fig. 2b). On the other hand, surface pressure over sub-polar low pressure belt centered around 60°N increased by about 1 mb, leading to weaker than normal low pressure belt. This reduction in meridional pressure gradient has strong implication on the wind (Fig. 2b, vectors), precipitation (Fig. 2c) and runoff (Fig. 2d) over North America and Europe. In the last 100 years of CR, there are 21 NAO+ years and 20 NAO− years, whereas there is a clear swing towards negative phase in AMZ during the same period, with 41 NAO− years and only 7 NAO+ years (Fig. 3a,b).

### Mechanism

**Figure 5:**
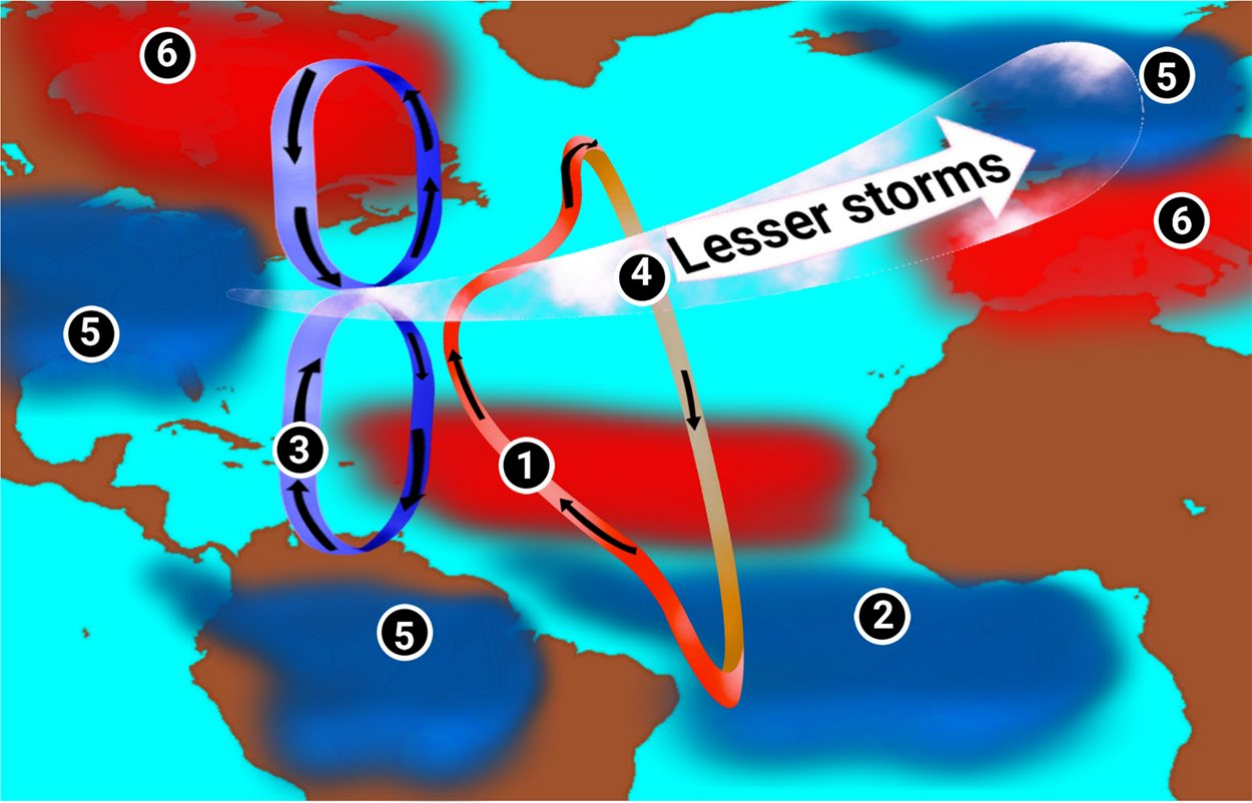
Teleconnection. Schematic showing how the shutdown of Amazon runoff into the Atlantic would lead to a shift in phase of NAO. Red shaded regions represent an increase whereas blue shaded regions show a decrease, compared to the CR simulation. Enumerations in the figure represent the order in which each parameter/region is affected. **(1)** Shutdown of Amazon runoff in the AMZ result in reinforced AMOC. **(2)** Stronger AMOC enhance southern tropical Atlantic upwelling and cooling. **(3)** Cooler equatorial Atlantic lead to a weakening of Atmospheric meridional cells. **(4)** Weaker meridional cells lead to sluggish extratropical westerlies, implying a negative phase of NAO. **(5)** Amazon basin, northern Europe, and the United States winter become drier and cooler. **(6)** Southern Europe and Canada turn warmer and wetter. This schematic was created using an open software Krita, Version 2.8.1 (https://krita.org/en). The background map used is generated using NCL.

The mechanism connecting Amazon runoff with NAO and the processes involved are summarized in Fig. 5. In the absence of Amazon runoff, surface ocean turns saltier in the northwestern tropical Atlantic (Fig. 1a). The mean poleward currents in the tropical northwest Atlantic (mainly Gulf Stream and North Brazilian Current) disseminate these SSS anomalies northward^12^. Saltier waters formed in the Amazon river mouth spread across the extratropical Atlantic within a decade of AMZ river runoff shut down. Compared to the OGCM study on effects of Amazon runoff^12^, the surface salinity anomalies are weaker, probably due to the negative feedback from the atmosphere. But the saltier and denser surface water in the AMZ could affect the sinking in the northern Atlantic; which is home for major deep-water formation sites. Thus the poleward transport of saltier water from Amazon river mouth results in an enhanced sinking in the extratropical Atlantic, reinforcing the AMOC^12^ (Fig. 1c). Sensitivity of AMOC to surface salinity fluctuations have been well documented in several freshwater sensitivity studies in the North Atlantic (for e.g. refs 13 and 20).

Associated with the strengthening of AMOC, the equatorward transport of cooler subsurface extratropical water enhances. Therefore, a reinforced ocean conveyor belt circulation^21^ is accompanied by strengthening of upwelling in the southern tropical Atlantic and enhanced northward cross-equatorial surface water transport^12, 22^. In the AMZ, the upwelling in the southeastern tropical Atlantic strengthens (Fig. 3c), bringing colder water to the surface which further intrudes into the northern equatorial Atlantic (Fig. 1a) via cross-equatorial currents^12, 22^. Slight warming to the north of 5°N is mainly driven by the atmospheric feedback via changes in local winds and air-sea fluxes (details in the Discussion section). Freshwater hosing experiments have affirmed that the robust response of tropical Atlantic to a stronger AMOC is the development of meridional SST dipole in the tropical Atlantic with a cooling to the south and a warming to the north of the thermal equator^23^ (Fig. 1a).

**Figure 4:**
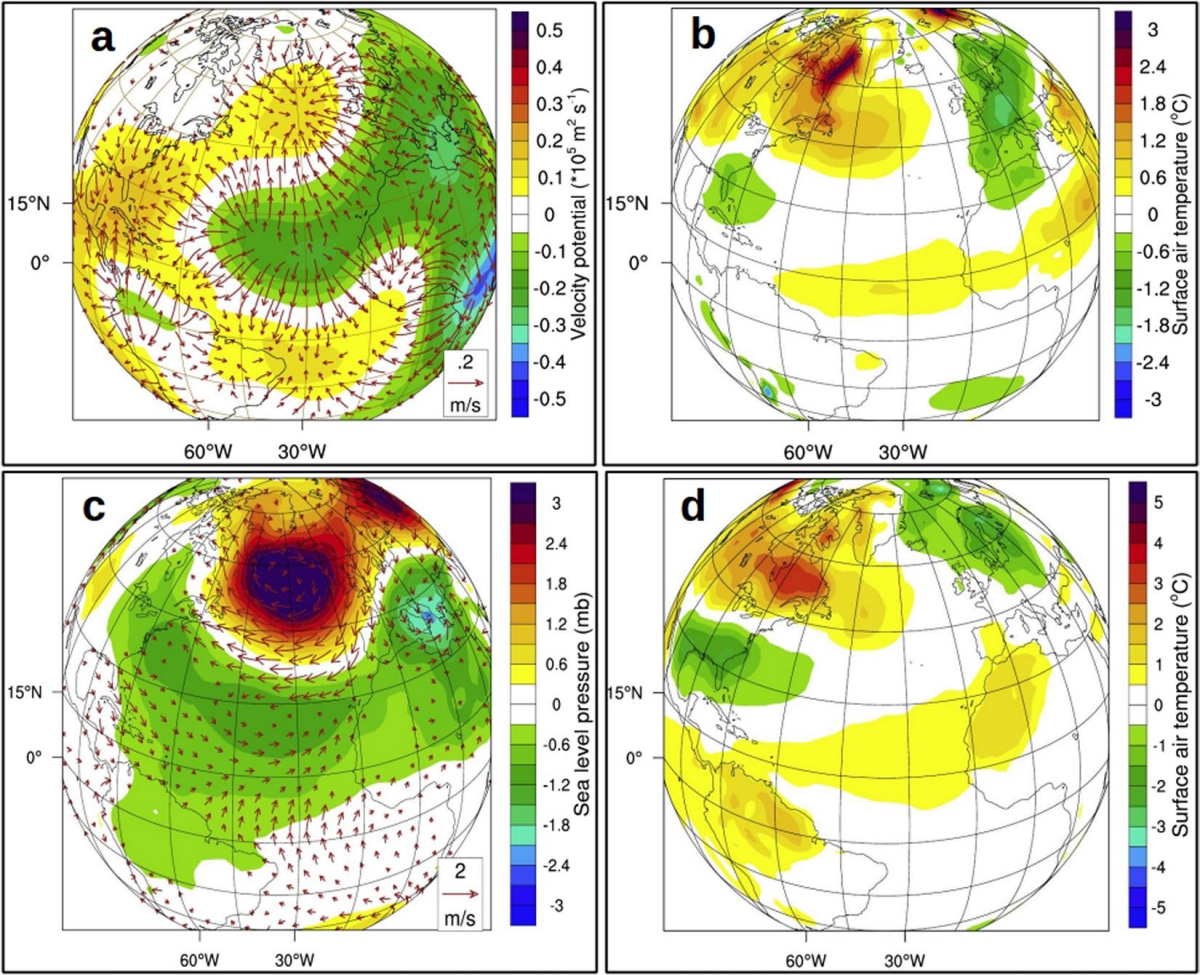
Negative phase of NAO and its impacts: II. **(a**) Mean DJF difference (AMZ-CR) in 500 mb omega (contour; Pa s^−1^) overlaid with divergent winds (vectors; m s^−1^) at the same level. A positive omega represents anomalous sinking and vice versa. (**b**) Composite of observed DJF surface air temperature (°C) anomaly during positive inter-hemispheric SST gradient (cooler South Atlantic; see methods). (**c**) Same as (**d**), but for SLP (mb) and winds (m s^−1^). (**d**) Composite of DJF surface air temperature (°C) during Amazon drought events. Plots were generated using an open software NCL (Version 6.3.0) [Software] (2016). Boulder, Colorado: UCAR/NCAR/CISL/TDD. http://dx.doi.org/10.5065/D6WD3XH5. Background map used is the satellite projection provided by NCL.

A positive dipole SST pattern in the tropical Atlantic (cooler to the south and warmer to the north) favors a more northerly position of ITCZ in the northern summer than the climatological positioning^24^. During winter, the cooling of surface ocean in the equatorial Atlantic weaken the local ascending (Fig. 3d) and thereby weaken the meridional pressure gradient and strength of the Hadley cell^25^. As a result, the upper tropospheric divergence over equatorial low and convergence over subtropical high deteriorates (vectors in Fig. 4a). The replication of these upper tropospheric changes on lower troposphere is a slow-down of divergent winds over subtropical high (implying weaker high pressure center; Fig. 4a) and anomalous weakening of the descending branch (centered around 28°N; Fig. 3d).

The difference in SLP in the northern Atlantic (Fig. 2b) shows that there is a weakening of subtropical high pressure belt with a maximum anomaly of 1.6 mb, centered around 50°N over western Europe. Lower than normal descend over the subtropical high pressure belt and weaker ascend over subpolar low pressure center (Fig. 3d) weaken the meridional pressure gradient in the extra-tropics. North Atlantic Oscillation, which is essentially the strength of meridional pressure gradient in the extratropical Atlantic, therefore, weaken in the absence of Amazon runoff (Fig. 3b). The strength of the westerlies in the northern Atlantic is primarily determined by the intensity of this meridional pressure gradient between subtropical high pressure belt and subpolar low pressure belt^16^. A negative (positive) phase of NAO is characterized by a lower (higher) than normal surface pressure over subtropical high pressure center and higher (lower) than normal pressure over the subpolar low, resulting in a weaker (stronger) than normal meridional pressure gradient^25^. The atmospheric and oceanic changes in the northern Atlantic and adjacent landmasses resemble a negative NAO-like climate (Fig. 2a–c). The significance of this change in phase of NAO is discussed below.

## Discussion

When the Amazon runoff is blocked, ocean responds by reinforcing AMOC, the outcome of which is a cooler than normal southern equatorial Atlantic. The intrusion of this cooler surface waters to the northern tropical ocean (up to ~5°N) weaken the northeasterlies (Fig. 2b) due to the reduction in SLP gradient between the equatorial low pressure belt and subtropical high pressure belt. Mild warming to the north of 5°N in the AMZ (Fig. 1a) is the outcome of wind-evaporation-SST feedback (Bjerknes feedback^26^) that suppress the local evaporative cooling (Fig. 1b) owing to the weakening of surface northeasterlies (Fig. 2b). This SST dipole in the tropical Atlantic reorganizes the surface easterly winds and currents, favoring the weakening of Atlantic ITCZ during winter (Fig. 2c)^24^.

The SST anomalies in the Atlantic interacts with the overlying atmosphere in the form of evaporation, precipitation, and sensible heat flux^27^. In the 0°N–20°N and 40°N–70°N latitude bands of tropical Atlantic Ocean, surface evaporation reduced by ~0.2 mm day^−1^ whereas there is higher evaporation of comparable magnitude centered around 30°N (Fig. 1b). Significant changes in wintertime sensible heat flux occurs in the northern Atlantic (40°N–70°N) with a gain (reduced loss of heat by the ocean) of 6 W m^−2^ in the western half and a loss of 5 W m^−2^ in the eastern half (Fig. 1b). Mechanism of SST changes in the extratropical Atlantic is, therefore, the outcome of complex air-sea interaction involving winds, evaporation and sensible heat flux. Changes in air-sea heat fluxes in the extratropical North Atlantic can also affect the strength of AMOC through changes upper ocean mixed layer depth and density^17^.

Tropical Atlantic rainfall response is the manifestation of underlying SST changes. Stronger (weaker) the meridional gradient of SST across the equator, weaker (stronger) will be the trades to the north (south) of equator^24^. The meridional position of Atlantic ITCZ shifts towards the warmest underlying ocean^25^. Cooler than normal surface waters in the southern equatorial Atlantic is accompanied by larger than normal northward migration in summer and weaker ITCZ during winter^24^. Thus cooling of underlying ocean weaken the local ITCZ in the AMZ during winter, lowering the local rainfall over Amazonia and equatorial Atlantic (Fig. 2c).

Associated with the negative NAO-like pattern in the AMZ, there are unique climatic features in the northern hemisphere, predominantly during winter (DJF). The sub-polar low pressure weakening has core over Greenland-Iceland-Norwegian Sea (GIN Sea, 60°N). The direct effect of this depreciated meridional SLP gradient is sluggish westerlies (lower by ~1 m s^−1^) in the extratropical Atlantic (Fig. 2b). Changes in strength and orientation of winds in the extratropical Atlantic alter the frequency and intensity of storminess in the northern Europe^14^. In response to the reorganization of winds in the extratropics, the ﻿northern United States and Canada experienced a warmer (by 0.6 °C) winter whereas Eurasia experienced a cooler (by 0.5 °C) winter (Fig. 2a). Arctic recorded warmer winters (mean of 0.4 °C) with a maximum increase of about 1.3 °C. Surface air temperature over Siberia and ﻿northeastern Russia cooled by more than 0.8 °C. Associated with the weakening of westerlies, northwestern Atlantic and the Greenland surface temperature is warmer^14^.

Changes in large-scale circulation pattern in the North Atlantic and adjacent landmasses have a substantial impact on northern hemispheric precipitation, north of 20°N^16^. Precipitation and temperature are lower than normal in northern Europe, including Iceland and Scandinavia (Fig. 2a,c); consistent with the climatic conditions during NAO−^28^. This is due to the weakening of westerlies (Fig. 2b) which considerably lowers the transport of moisture to the northern Europe (south of 60°N) and hence a drier than normal climate (Fig. 2c). Southern Europe (south of 60°N), on the other hand, experiences a wetter than normal atmospheric condition^16, 28^. Cooler and drier Siberia is also associated with pressure fluctuations in north Atlantic^14, 16^. Warmer northwestern Atlantic and adjacent landmasses including northern Canada (Fig. 2a) resulted in wetter than normal climate in the northern Canada and northeastern United States (Fig. 2c); in agreement with the changes during NAO−^14, 16^. The weakening of the Atlantic ITCZ resulted in reduced runoff over Amazonia whereas the Mississippi river runoff increased, consistent with rainfall anomalies over land (Fig. 2d). Most of the European rivers running into the Atlantic flooded in the absence of Amazon runoff and the pronounced warmer winter temperature over Arctic resulted in a reduction in the percentage of sea-ice aggregate area (Fig. 2d).

Interestingly, some of the atmospheric proxies based on observations (see methods) yield responses similar to our experiments. The composite of surface air temperature (Fig. 4b), SLP and surface wind anomalies (Fig. 4c) during positive equatorial Atlantic meridional SST gradient (see methods) exhibit a strikingly similar large-scale pattern as that of AMZ anomalies (Fig. 2a,b) in the extratropical northern hemisphere. Observed composites of surface air temperature show that northern Europe and the eastern United States experienced cooler winters whereas Canada turned warmer (Fig. 4b) when the observed tropical Atlantic SST gradient is positive, similar to the atmospheric response in the absence of Amazon river runoff (Fig. 2a). In short, factors responsible for altering the meridional thermal seesaw in the tropical Atlantic have the potential to determine the phase of NAO; Amazon runoff being a prominent one. Composite of observed surface air temperature anomaly (Fig. 4d; see methods) during historical lows in the Amazon runoff matches well with the large-scale AMZ anomaly pattern (Fig. 2a). An important factor to note here is that, apart from the effect of El Niño, the Amazon drought events can also be caused by cooling of equatorial Atlantic Ocean. The anomalous negative NAO-like surface air temperature anomaly observed in Fig. 4d could be an after-effect of this cooling of equatorial waters. Here we suggest that significant changes in the runoff can also result in cooling of equatorial Atlantic which in turn is responsible for weaker NAO. Thus, the scenario of drying up of Amazon runoff could lead to large shifts in the mean climate over Europe and North America.

**Figure 6:**
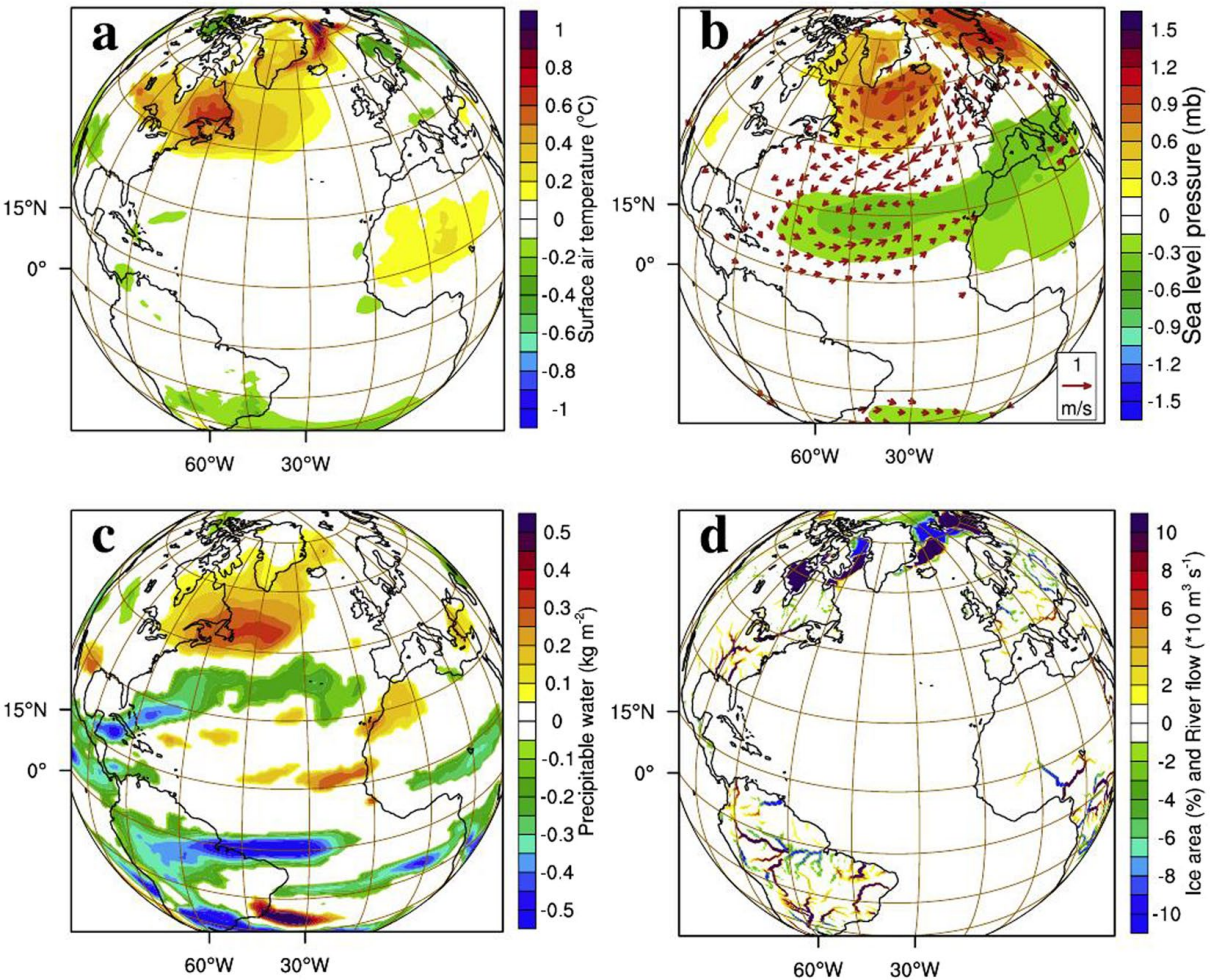
Results from tropical Atlantic experiment (TropAtl). Mean DJF difference (TropAtl-CR) in (**a**) surface air temperature (°C), (**b**) sea level pressure (mb) and winds (m s^−1^) with reference vector of 1 m s^−1^, (**c**) precipitable water (kg m^−3^) and (**d**) river runoff (×10^−1^ m^3^ s^−1^) in the land model and percentage change in ice area (%). Shaded regions are significant at 90% confidence level using t-test. Plots were generated using an open software NCL (Version 6.3.0) [Software] (2016). Boulder, Colorado: UCAR/NCAR/CISL/TDD. http://dx.doi.org/10.5065/D6WD3XH5. Background map used is the satellite projection provided by NCL.

An additional simulation was carried out to assess the combined climatic impact of the shutdown of all the major tropical Atlantic rivers (TropAtl). In TropAtl, Amazon, Congo, and the Orinoco river runoff into the tropical Atlantic Ocean were blocked (see methods). Atmospheric response to the absence of tropical Atlantic rivers is similar to that of AMZ simulation. Anomalies of surface air temperature (Fig. 6a), sea level pressure and wind (Fig. 6b), precipitable water (Fig. 6c), and runoff and snow cover (Fig. 6d) are similar to AMZ anomalies, but with a weaker magnitude. A possible reason for this milder response could be due to the fact that the SST anomaly generated in TropAtl simulation is weaker than AMZ due to weaker cross-equatorial meridional salinity gradient, when the Congo river is shut down.

This study reaffirms the role of tropical Atlantic SST on the NAO variability through changes in atmospheric meridional cells triggered by the weakening of ITCZ (summarized in Fig. 5). Model simulations suggest that the reduction in runoff into the tropical Atlantic have a significant impact on the global water cycle and large-scale climate pattern, namely NAO. Substantial reduction in the Arctic sea-ice extent and changes in river flow into the oceans in the extratropical Atlantic could change the upper ocean stratification and therefore modulate the deep-water formation. Moreover, a considerable reduction in Amazon runoff could further reduce rainfall over the Amazonia, affecting the global carbon and freshwater cycle. Surface temperature changes in the tropical Atlantic generated by turning off Amazon river runoff can be responsible for extensive changes in climate over northern Atlantic and adjacent landmasses, through atmospheric pathways. In the wake of this result, we call for a proper scientific understanding of the climatic role of Amazon runoff while preparing conservation framework for Amazon ecosystem.

### Model details

Community Earth System Model (CESM version 1.2^29^), with active atmosphere, ocean, land, sea ice and runoff components has been used to assess the climatic role of freshwater influx into tropical Atlantic. The atmospheric component of CESM is Community Atmospheric Model (CAM version 5^30^) with a finite volume dynamical core at a horizontal resolution of 0.47° × 0.23° with 30 vertical levels discretized using a hybrid sigma-pressure coordinates. Community Land Model (CLM version 4^31^) which has a carbon-nitrogen cycle incorporated in it, shares the same horizontal grid with the atmosphere with 15 soil layers in the vertical. The ocean model employed is Parallel Ocean Model (POP, version 2^32^) that uses a displaced pole horizontal grid and a z-coordinate vertical grid with a longitudinal resolution of ~1° and a latitudinally varying resolution, with highest resolution near the equator (0.27°) and coarsest near the poles (1.0°). There are 60 levels in the vertical with a grid spacing of 5 m in the top 150 m and gradually increasing towards the bottom. River Transport Model (RTM^33^) is used to direct surface runoff from land to active ocean or marginal sea. Model control run (CR) simulates pre-industrial (CO_2_ concentration of 284.7 ppmv) climate for a period 200 years, started from spun-up restart files provided by National Center for Atmospheric Research (NCAR).

### Amazon runoff Experiment

The experiment without Amazon runoff (AMZ) is carried out for the same period with same initial conditions as that of CR by setting the runoff flux transferred from coupler to the ocean to zero over the Amazon river mouth (Fig. 1a). Last 100 years of the model output from CR and AMZ experiments are used for analysis. Results from the AMZ simulates an ideal climate in which prolonged drought would result in Amazon river running dry throughout the year. The mean difference in oceanic and atmospheric fields of the AMZ compared to the CR is considered as the climatic impacts of Amazon runoff.

### Tropical Atlantic runoff Experiment

In tropical Atlantic runoff experiment (TropAtl), all rivers streaming into the tropical Atlantic in the band of 20°S to 10°N is shut off. Major rivers excluded in this experiment are Amazon, Congo, and Orinoco. Similar to AMZ experiment, last 100 years of TropAtl simulation is used for the analysis. This experiment demonstrates the combined effect of all the tropical Atlantic rivers on climate.

### North Atlantic Oscillation index

The phase of NAO is determined by an index that represents the 4-year running average of the normalized difference in surface pressure over subtropical high pressure center (35°N–55°N, 20°W–20°E) and subpolar low pressure center (60°N–80°N, 20°W–20°E) in the Atlantic. DJF pressure gradient with standard deviation (SD) greater than one are identified as positive NAO years and less than one SD are listed as negative NAO years.

### Observational Datasets used

SST data used in Fig. 4b,c is taken from Centennial *in situ* Observation-based Estimates (COBE-SST). The inter-hemispheric SST gradient is calculated as the area average SST difference between northern tropical Atlantic (6°N–15°N) and southern tropical Atlantic (20°S–6°N). Surface air temperature, wind, surface pressure and precipitation data used in Fig. 4b–d for the period 1948 to 2000 is obtained from National Centers for Environmental Prediction (NCEP) reanalysis. Here, Amazon drought events are identified as the years where the river runoff into the Amazon is less than one SD of the mean runoff. Amazon river runoff data from Dai and Trenberth dataset^1^ for the period 1948 to 2000 is used to estimate drought years in Fig. 4d.

### Data Availability

Model output data will be made available to researchers upon request. Observation datasets used in this study can be downloaded from Dai and Trenberth runoff data set (www.cgd.ucar.edu/cas/catalog/surface/dai-runoff/) NCEP (www.esrl.noaa.gov/psd/data/gridded/data.ncep.reanalysis.derived.surface.html), and COBE-SST (www.esrl.noaa.gov/psd/data/gridded/data.cobe.html).
